# Late‐life mood disorder as the initial presentation of progressive supranuclear palsy: A case series

**DOI:** 10.1002/pcn5.178

**Published:** 2024-02-21

**Authors:** Kei Ichijo, Keisuke Takahata, Shin Kurose, Takemi Watanabe, Yukihiro Nagase, Hironobu Endo, Kenji Tagai, Makoto Ishitobi, Makoto Higuchi

**Affiliations:** ^1^ Takatsuki Hospital Hachioji Tokyo Japan; ^2^ Department of Functional Brain Imaging, Institute for Quantum Medical Science, Quantum Life and Medical Science Directorate National Institutes for Quantum Science and Technology Inage Chiba Japan; ^3^ Department of Psychiatry Nihon University School of Medicine Itabashi Tokyo Japan; ^4^ Jinkei Hospital Himeji Hyogo Japan

**Keywords:** late‐life mood disorder, progressive supranuclear palsy (PSP), tauopathy

## Abstract

**Aim:**

Progressive supranuclear palsy (PSP) is a rapidly progressive neurodegenerative disorder characterized by Parkinsonism, supranuclear ophthalmoplegia, postural instability, and cognitive impairment.

**Patients:**

This case series describes three patients initially diagnosed with late‐life mood disorders (depression and bipolar disorder) who were later diagnosed with PSP because of the development of typical neurological symptoms.

**Result:**

The diagnostic challenge of PSP is highlighted in this case report, particularly in the early stages, when characteristic symptoms may not be present. The importance of considering PSP in the differential diagnosis of late‐life mood disorders, especially in the absence of response to standard antidepressant therapy, is also emphasized. The heterogeneity of PSP is described, with various subtypes and atypical variants presenting with different clinical features. The psychiatric symptoms of PSP include apathy, disinhibition, depression, and anxiety, whereas hallucinations and delusions are less frequent. Tau positron emission tomography imaging is discussed as a potential biomarker for atypical PSP.

**Conclusion:**

Early diagnosis and intervention are crucial for improved outcomes in PSP, necessitating further research to enhance the diagnostic and treatment strategies for PSP and other neurodegenerative diseases.

## Graphical abstract

Progressive supranuclear palsy (PSP) is a rapidly progressive neurodegenerative disorder characterized by Parkinsonism, supranuclear ophthalmoplegia, postural instability, and cognitive impairment. This case series describes three patients initially diagnosed with late‐life mood disorders (depression and bipolar disorder) who were later diagnosed with PSP due to the development of typical neurological symptoms.

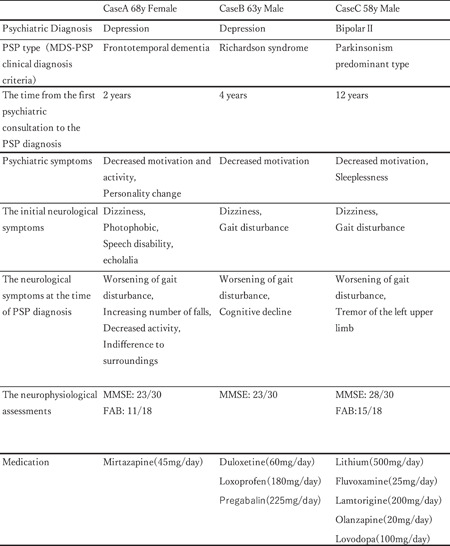

## INTRODUCTION

Progressive supranuclear palsy (PSP) belongs to the frontotemporal lobar degeneration‐tau (FTLD‐tau) spectrum disorders.^1^ The clinical features include Parkinsonism, supranuclear ophthalmoplegia, postural instability, and cognitive impairments. These symptoms usually appear between the ages of 55 and 70 years and are characterized by rapid progression.^2^ Although a definitive diagnosis of PSP requires neuropathological examination, the Movement Disorder Society PSP diagnostic criteria are widely used for antemortem PSP diagnosing.^3^ However, the characteristic symptoms may not always be present in the early stages of the disease, making the task of clinical diagnosis arduous.

PSP is considered to be a pathological continuum from a presymptomatic phase to a fully symptomatic phase through a suggestive phase.^4^ In most cases during these clinical phases, the symptoms meet the clinical diagnostic criteria for possible or probable PSP–Richardson's syndrome or a variant PSP syndrome. However, in recent years, cases with a variety of atypical symptoms, including psychiatric symptoms, have been reported.^5^ Moreover, it is increasingly recognized that there are various forms of the disease other than the typical clinical form.^4^ The most frequent psychiatric symptoms reported in patients with PSP are apathy and disinhibition, with depression and anxiety also being commonly elicited.

In contrast, hallucinations and delusions, such as those seen in patients with Parkinson's disease (PD), are less frequent.^6^ Previous studies have shown that patients with advanced PSP consistently experience more severe affective and cognitive symptoms, including apathy, depression, and executive and visual–spatial deficits, in comparison to patients with PD.^7^ Nevertheless, few cases have been reported in which psychiatric symptoms were the primary initial symptoms of PSP.^8^ Epidemiological studies have reported that late‐life mood disorders are associated with an increased risk of neurodegenerative diseases.^9^ Consistently, recent positron emission tomography (PET) studies have shown that tauopathies are involved in the pathogenesis of late‐life psychiatric disorders.^10^ These tauopathies include Alzheimer's disease (AD) and various non‐AD tauopathies. However, few reports have described the longitudinal course of change in clinical diagnosis from late‐life psychiatric disorders to neurodegenerative diseases, particularly PSP.^8^

Considering the heterogeneity in the pathophysiology of late‐life mood disorders, clinical reports on the longitudinal course of patients would be of significant value.

In this case series, we describe three patients who were initially diagnosed with late‐life mood disorders and later with PSP. All patients initially presented with mood disorders, such as depression and bipolar disorder, but the diagnosis was later changed to PSP because of the development of typical neurological symptoms.

## METHODS

### Patients

We conducted longitudinal clinical and neuroimaging assessments of the three patients. Magnetic resonance imaging (MRI) data were obtained for each case, and PET data were obtained for Case C. Written informed consent was obtained from all the participants. The Institutional Review Board of the National Institute for Quantum Science and Technology, Chiba, Japan, approved the study protocol.

### PET scanning

We performed PET imaging using ^11^C‐Pittsburgh Compound‐B (^11^C‐PiB) and ^18^F‐florzolotau (^18^F‐PM‐PBB3/^18^F‐APN‐1607) to examine the presence of pathological protein aggregates. We calculated the standardized uptake value ratios (SUVRs) of ^18^F‐florzolotau retention using reference voxels extracted from gray matter segments^11^ to quantify tau accumulations, considering the possibility that no brain areas without tau deposits were available as a reference region.

## RESULTS

### Case description

#### Case A

A 68‐year‐old female with no past or family history of psychiatric or neurological disorders presented to an outpatient general medicine clinic with sadness, loss of interest, anxiety, and depression. Since various physical examinations failed to reveal abnormalities, the patient was referred to a psychiatrist. She was diagnosed with major depressive disorder without psychotic symptoms and prescribed antidepressants. However, her symptoms did not improve.

Seven months after the visit to the psychiatric clinic (approximately 1 year after the onset of symptoms), she was referred to our clinic for consultation. Then, the dose of the antidepressant was increased (15 mg/day mirtazapine to 30 mg/day) (Table 1), but her symptoms did not improve.

**Table 1:** Clinical characteristics of three progressive supranuclear palsy cases.

	Case A: 68‐year‐old female	Case B: 63‐year‐old male	Case C: 58‐year‐old male
Psychiatric diagnosis	Depression	Depression	Bipolar Ⅱ
PSP type (MDS‐PSP clinical diagnosis criteria)	Frontotemporal dementia	Richardson syndrome	Parkinsonism predominant type
Time from the first psychiatric consultation to the PSP diagnosis	2 years	4 years	12 years
Psychiatric symptoms	Decreased motivation and activity, personality change	Decreased motivation	Decreased motivation, sleeplessness
Initial neurological symptoms	Dizziness, photophobic, speech disability, echolalia	Dizziness, gait disturbance	Dizziness, gait disturbance
Neurological symptoms at the time of PSP diagnosis	Worsening of gait disturbance, increasing number of falls, decreased activity, indifference to surroundings	Worsening of gait disturbance, cognitive decline	Worsening of gait disturbance, tremor of the left upper limb
Neurophysiological assessments	MMSE: 23/30, FAB: 11/18	MMSE: 23/30	MMSE: 28/30, FAB:15/18
Medication	Mirtazapine (45 mg/day)	Duloxetine (60 mg/day)	Lithium (500 mg/day)
		Loxoprofen (180 mg/day)	Fluvoxamine (25 mg/day)
		Pregabalin (225 mg/day)	Lamotrigine (200 mg/day)
			Olanzapine (20 mg/day)
			Levodopa (100 mg/day)

Abbreviations: FAB, Frontal Assessment Battery; MDS, Movement Disorder Society; MMSE, Mini Mental State Examination; PSP, progressive supranuclear palsy.

Approximately 4 months after attending the Takatsuki outpatient clinic, she developed photophobia, dizziness, and decreased language fluency. In addition, behavioral problems reminiscent of apraxia, such as failure to hold a kettle and burns, inability to put on slippers while standing, inability to hang pants on a hanger, and inability to tie shoelaces, appeared occasionally. She was then referred to the Department of Psychiatry at Nihon University Itabashi Hospital and admitted for further examination and diagnosis. Here, neurological examination showed eye movement disorders. Moreover, upward rotation, postural reflex disorder, and applause tests were positive. Furthermore, she exhibited faltered speech with syllabic stress and was suspected of having speech apraxia.

Trail B and Stroop tests were more than 1.5 SD below the corrected values.

Frontal Assessment Battery (FAB) score was 11/18, and applause signs were positive.^12^ These findings demonstrated frontal lobe dysfunction.

**Figure 1:**
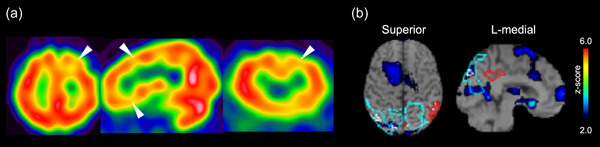
Cerebral perfusion single photon emission computed tomography (SPECT) images of Case A. (a) Images of brain perfusion SPECT of Case A. Colored areas show significantly increased (red) or decreased (blue) perfusion compared with (b) age‐matched controls.

MRI demonstrated mild frontal lobe atrophy. Brain perfusion single photon emission computed tomography (SPECT) showed decreased blood flow in the frontal lobe (Figure 1). Based on these clinical and neuroimaging assessments, the patient was diagnosed with PSP (frontal lobe symptom‐predominant type).

**Figure 2:**
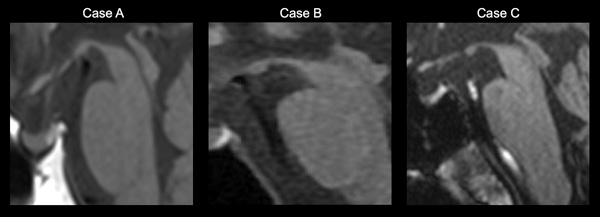
Midbrain atrophy in progressive supranuclear palsy (PSP) cases. Midsagittal T1‐weighted images in PSP patients with increasing midbrain atrophy (Cases A, B, and C).

Six months later, MRI showed progressive atrophy of the frontal lobes and midbrain capsule (hummingbird sign) (Figure 2). The patient began to repeat the same words frequently during conversations (tautology). Moreover, her interest and spontaneous activities decreased markedly. She was admitted to Takatsuki Hospital because of the difficulty of living at home because of repeated falls. Subsequently, she was admitted to a special nursing home because of progressive cognitive decline. According to a letter from her husband, she had recurrent aspiration pneumonia and died at the age of 72 years.

#### Case B

A 63‐year‐old male with depression attempted suicide and suffered a cervical spinal cord injury. The only organic abnormality was the cervical spinal cord injury, and no intracranial abnormalities were observed. After his physical recovery, he was examined by a psychiatrist, who prescribed antidepressants (20 mg/day duloxetine). The patient was referred to Takatsuki Hospital for treatment of depression.

We continued the antidepressant medication (20 mg/day duloxetine). Because it did not change his symptoms, such as decreased motivation and depression, the dose was increased to 60 mg/day, but no significant improvement was observed.

The patient had numbness in his hands and feet because of cervical spinal cord injury, and the analgesics (loxoprofen 180 mg/day, pregabalin 225 mg/day) prescribed by the orthopedic surgeon were continued concomitantly (Table 1).

Two years later, the patient complained of numbness and pain in his hands and feet, along with dizziness and lightheadedness. One year later, he had difficulty walking, fell down some stairs, and suffered a compression fracture of the lumbar vertebrae. It was difficult for him to stay home, and he was admitted to Takatsuki Hospital.

His gait deteriorated approximately 4 years after he began attending Takatsuki outpatient clinic. His cognitive function decreased, and he was transferred to the National Center of Neurology and Psychiatry for a thorough examination. Neurological examination revealed limited upward eye movements, predominant muscle stiffness in the right upper extremity, and mild frontal lobe signs. An MRI of the brain revealed midbrain atrophy and hummingbird signs (Figure 1). Brain SPECT revealed decreased blood flow in the frontal lobe.

Based on these results, the patient was diagnosed with PSP (Richardson type).

Subsequently, he fractured his pelvis because of easy falls, and his Activities of Daily Living were reduced because of wheelchair dependency. Dysarthria and dysphagia developed, and aspiration pneumonia recurred. Gastroparesis was induced as oral intake became more difficult. Two years after the diagnosis of PSP, the patient died because of aspiration pneumonia.

#### Case C

A 58‐year‐old male presented at Takatsuki Hospital with depression and anxiety. Eleven years previously, he had been diagnosed with cancer of the left renal plevis and had undegone a nephrectomy. (Table 1). After the surgery, depression and anxiety developed, and he started visiting a psychiatrist. Six years later, he became busy at work, started to feel elated, and engaged in activities. Subsequently, he experienced a decline in motivation and felt anxious. He was diagnosed with a depressive episode of bipolar II disorder, and treatment with 200 mg/day of lithium, 200 mg/day of lamotrigine, and 25 mg/day of fluvoxamine by his previous physician commenced. Since there was no improvement in depressive symptoms, we discontinued fluvoxamine. As maintenance therapy for bipolar disorder, 10 mg/day of olanzapine was added.^13^, ^14^ Despite these treatments, mild depressive symptoms persisted without improvement (Table 1).

When gait disturbance appeared, it was considered to be a side‐effect of lithium, which had been prescribed to the patient by his previous doctor. Therefore, lithium was tapered from 200 to 100 mg/day. However, his physical symptoms did not improve, and he was referred to Takatsuki Hospital. We first tapered off the lithium. However, the gait disturbance worsened, and tremors localized in the left upper limb appeared.

The patient was referred to the Department of Neurology at the Municipal Medical Center for further evaluation of gait disturbances and tremors. Neurological findings were as follows. He had a mask‐like appearance and noticeably slower movements. No clear rigidity and a localized tremor were observed in the left upper extremity. He had a forward‐leaning gait, a scuffling gait, and a slight wiggle. Backward thrusting was negative. Eye movements were unremarkable. MRI showed atrophy with frontal dominance, and the sagittal section showed atrophy of the midbrain (hummingbird sign) (Figure 1).

Dopamine transporter scintigraphy showed decreased accumulation, whereas meta‐iodobenzylguanidine scintigraphy did not exhibit decreased accumulation. Therefore, PD was ruled out. Based on the abovementioned findings and laboratory investigation results, a diagnosis of PSP‐P (Parkinsonism predominant type) was made.

**Figure 3:**
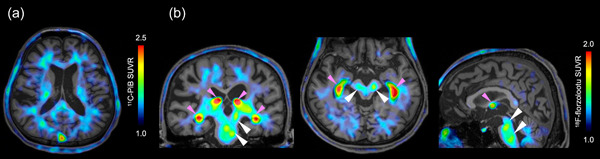
Amyloid and tau positron emission tomography (PET) findings of Case C. Images of (a) amyloid PET with 11C‐PiB and (b) tau PET with 18F‐florzolotau in Case C. White triangles indicate brain regions with tau accumulation, and off‐target binding in the choroid plexus is indicated by pink triangles.

We performed a tau PET study with ^18^F‐florzolotau at the National Institute for Quantum Science and Technology. The findings of PET imaging were consistent with those for early PSP and PSP‐P (Figure 3). Levodopa was prescribed for the tremor in the left upper extremity, and temporary improvement was observed. The addition of levodopa did not cause any relapse or worsening of manic or other symptoms. He continues to be an outpatient, but his gait disturbances and tremors have gradually worsened.

The clinical characteristics of each case are summarized in Table 1.

## DISCUSSION

This report describes three cases that highlight the diagnostic challenge of PSP, particularly in its early stages when characteristic symptoms may not be present. Each case had a prolonged duration between initial presentation and PSP diagnosis, during which the patients received treatment for depression or bipolar disorder. In all three cases, the development of neurological symptoms, such as gait disturbances and cognitive decline, eventually led to a diagnosis of PSP. These findings emphasize the importance of considering PSP in the differential diagnosis of late‐life mood disorders and other psychiatric symptoms, particularly when standard antidepressant therapy fails to show any improvement. Further studies are warranted for a better understanding of the relationship between PSP and psychiatric disorders and to develop more effective diagnostic and treatment strategies for PSP.

The three cases presented different PSP subtypes, demonstrating the disease's heterogeneity. Although classical PSP–Richardson's syndrome and PSP‐P are the most common subtypes, there have been increasing instances of atypical variants with varying clinical presentations.^3^ The most frequent psychiatric symptoms in PSP are apathy and disinhibition. Moreover, depression and anxiety are also often present. In contrast, hallucinations and delusions, such as those observed in PD, are less frequent.^6^ Patients with advanced stages of PSP consistently suffer from more severe affective and cognitive symptoms, including apathy, depression, and executive and visual–spatial deficits, in comparison to patients with PD.^15^

It is important to distinguish depression from apathy in late‐life mood disorders. Apathy is a widely recognized psychiatric symptom of PSP and other neurodegenerative diseases.^16^ It is characterized by a decrease in goal‐directed behavior in terms of behavioral, cognitive, emotional, and social aspects, with a neutral mood and thinking rather than negative thinking.^17^ Superficially, apathy can be misdiagnosed as it often resembles depression. Symptoms such as sadness, self‐doubt, loss of self‐confidence, decreased motivation, and dread may appear in depression. Physical symptoms, such as decreased appetite, weight loss, sweating and flushing, and palpitations, along with daily fluctuations, with the morning being the most sluggish, are also often present in depression. In contrast, motor symptoms rarely precede apathy, and psychiatric symptoms are characterized by the absence of sadness and remorse.^18^ These distinctions are important in differentiating apathy from depression. Only a few cases of depression preceding the onset of PSP that have been psychiatrically treated have been reported.^8^, ^19^

The prevalence of depression in PSP and other neurodegenerative diseases, such as corticobasal degeneration (CBD) and PD, is also noteworthy. Depression is a common non‐motor symptom in both diseases and is associated with increased morbidity and mortality rates.^20^ A study reported that depression was present in 20%–40% of patients with PD^21^ and 73% of patients with CBD.^22^ Furthermore, a meta‐analysis of 122 autopsy cases of PSP reported that 4.1% of the deceased were diagnosed with depression at the time of initial diagnosis.^23^ Therefore, it is important to consider the possibility of underlying neurodegenerative diseases in patients presenting with late‐life depression or other psychiatric symptoms, especially in those with atypical features or lack of response to standard treatment. Case C is characterized by a brief hypomanic episode followed by prolonged depression. Such transient manic episodes must be differentiated from the symptoms seen in PSP depression.

One of the patients in this study underwent tau PET scanning, which showed an increased accumulation of tau in the midbrain and subthalamic nucleus, consistent with early PSP and PSP‐P. Using tau PET as a biomarker can help diagnose PSP with atypical symptoms,^24^ particularly in cases where the clinical presentation is atypical or diagnostic uncertainty exists. However, tau PET is not widely available, and further studies are needed to validate its utility in diagnosing and managing PSP.

This study had one limitation: Since no autopsies were performed, the neuropathological findings of PSP were not confirmed in the postmortem brains of the patients.

In conclusion, the cases presented in this report highlight the importance of considering PSP as a possible diagnosis for patients presenting with late‐life mood disorders, particularly in the absence of a response to standard antidepressant therapy. Early diagnosis and intervention may improve the outcomes in patients with PSP. Further studies are needed for a better understanding of the relationship between PSP and psychiatric disorders and to develop more effective diagnostic and treatment strategies for PSP and other neurodegenerative diseases.

## AUTHOR CONTRIBUTIONS

All authors contributed to the study's conception and design. Kei Ichijo, Keisuke Takahata, Shin Kurose, Takemi Watanabe, Yukihiro Nagase, Hironobu Endo, Kenji Tagai, Makoto Ishitobi, and Makoto Higuchi: data collection and analysis. Kei Ichijo and Keisuke Takahata wrote the first draft of the manuscript. All authors commented on previous versions of the manuscript. All authors read and approved the final manuscript.

## CONFLICT OF INTEREST STATEMENT

Keisuke Takahata is an Editorial Board member of *Psychiatry and Clinical Neurosciences Reports* and a co‐author of this article. To minimize bias, they were excluded from all editorial decision‐making related to the acceptance of this article for publication. The remaining authors declare no conflict of interest.

## ETHICS APPROVAL STATEMENT

The Institutional Review Board of the National Institute for Quantum Science and Technology, Chiba, Japan, approved the study protocol.

## PATIENT CONSENT STATEMENT

Written informed consent was obtained from all the participants.

## CLINICAL TRIAL REGISTRATION

N/A.

## Data Availability

The data supporting this study's findings are available on request from the corresponding author. The data are not publicly available due to their containing information that could compromise the privacy of research participants.
